# Molecular Mechanisms Underlying Recurrence in Triple-Positive Breast Cancer (ER+/PR+/HER2+) and Potential Repurposing of Multi-Target Inhibitors

**DOI:** 10.3390/ijms27125440

**Published:** 2026-06-16

**Authors:** Cindy Bandala, José Anselmo López-Méndez, María R. J. Díaz-Rivera, Jazmín Carro-Rodríguez, Martiniano Bello

**Affiliations:** 1 Laboratorio de Medicina Traslacional, Sección de Estudios de Posgrado e Investigación, Escuela Superior de Medicina, Instituto Politécnico Nacional, Plan de San Luis y Salvador Diaz Mirón s/n, Casco de Santo Tomás, Miguel Hidalgo, Mexico City 11340, Mexicojcarror2400@alumno.ipn.mx (J.C.-R.); 2 Laboratorio de Diseño y Desarrollo de Nuevos Fármacos e Innovación Biotecnológica, Sección de Estudios de Posgrado e Investigación, Escuela Superior de Medicina, Instituto Politécnico Nacional, Plan de San Luis y Salvador Diaz Mirón s/n, Casco de Santo Tomás, Miguel Hidalgo, Mexico City 11340, Mexico; hanselop6@iibiomedicas.unam.mx (J.A.L.-M.); mdiazr2500@alumno.ipn.mx (M.R.J.D.-R.)

**Keywords:** molecular mechanism, recurrence, triple-positive breast cancer, repurposing, multi-target inhibitors

## Abstract

Triple-positive breast cancer (TPBC) is characterized by the overexpression of estrogen receptor (ER), progesterone receptor (PR), and human epidermal growth factor receptor 2 (HER2), making its management a therapeutic challenge. Despite the availability of targeted therapies, patients with TPBC often experience recurrence and poor clinical outcomes due to intrinsic and acquired resistance mechanisms. This review summarizes current therapeutic approaches and their limitations, highlights the molecular mechanisms underlying treatment resistance and recurrence, and explores opportunities for drug repurposing, particularly involving multi-target inhibitors. Special emphasis is placed on the interaction between hormone receptor and growth factor receptor pathways, compensatory signaling mechanisms, and predictive biomarkers of recurrence. Furthermore, emerging strategies for drug repurposing using clinically available drugs are analyzed, including in silico, in vitro, and clinical trial evidence, along with their translational implications. Finally, we conclude that drug repurposing and multi-target approaches offer a compelling rationale for the development of novel therapeutic strategies in triple-positive breast cancer. However, their clinical utility remains to be validated through appropriately designed experimental and clinical studies before their impact on recurrence outcomes can be established.

## 1. Introduction

Breast cancer (BC) is the most common cancer type in women worldwide and continues to be one of the leading causes of cancer-related mortality [1]. Despite advances in prevention, early diagnosis, and therapeutic strategies, BC is expected to double its incidence and mortality by 2050 [2]. Technological advances in gene expression profiling, such as microarray analysis, as well as studies conducted by Perou [3] and Sørlie [4], have demonstrated that BC is a genetically heterogeneous disease. Clinically, BC is classified as luminal A (high ER and PR expression), luminal B (high ER, low PR and variable HER2 expression), HER2-enriched (lacking ER and PR, with high HER2 expression), and triple-negative (lacking ER, PR, and HER2 expression) [5]. This classification has enabled the development of therapeutic strategies, such as those based on the guidelines of the National Comprehensive Cancer Network, which have improved patient survival. Nevertheless, a significant proportion of patients, particularly those with clinically aggressive tumors such as HER2-positive and triple-negative BC, experience relapses following treatment [6].

TPBC is generally defined as a subtype of BC that expresses ER, PR, and HER2. TPBC accounts for approximately 15% of BC cases [7,8,9] and is influenced by the activation and interconnection of ER, PR, and HER2 signaling pathways. These interactions result in distinct clinical and pathological features compared to other subtypes, including large, calcified tumor masses with irregular margins and vascular or neural invasion. These tumors are often classified as grade III [10] and present a therapeutic challenge due to their high heterogeneity in mutations and gene expression profiles [5].

Current treatment strategies for TPBC typically combine chemotherapy, HER2-targeted therapy, and endocrine therapy, with the specific therapeutic regimen depending on the disease stage and clinical setting (neoadjuvant, adjuvant, or metastatic disease) [11]. Despite these advances, TPBC remains a clinically challenging subtype, as patients exhibit a higher risk of recurrence and poorer survival outcomes than those diagnosed with hormone receptor-positive/HER2-negative BC [10,11]. Furthermore, TPBC displays distinct gene expression and phosphoproteomic profiles associated with therapeutic resistance compared with luminal A and HER2-enriched breast cancer subtypes [2,9,12].

In the search for new therapeutic agents to counteract drug resistance in TPBC, drug repurposing could be a rapid and inexpensive alternative. Drug repurposing involves using existing drugs to treat diseases or conditions other than those for which they were originally developed [13,14]. Therefore, the general objective of this review is to provide an overview of the protein modifications and/or alterations present in TPBC resistant to therapy that could serve as potential targets for repurposed drugs.

## 2. Methodology

We conducted a narrative review of TPBC, focusing on its molecular resistance mechanisms and potential treatments to reduce recurrence. We included information from the following sources: PubMed, Web of Science, Scopus, ScienceDirect, SciFinder, ProQuest, EBSCO, Google Scholar, ClinicalTrials.gov, PubChem, NCBI Bookshelf, DrugBank, and LiverTox. After analyzing the information, we considered original research, narrative reviews, systematic reviews, meta-analyses, and progress reports. The search strategy incorporated combinations of keywords such as the following: “triple-positive breast cancer” OR “ER+/PR+/HER2+ breast cancer” AND “therapeutic resistance”, “drug resistance”, “recurrence”, “molecular mechanisms”, “HER2 signaling”, “estrogen receptor signaling”, “progesterone receptor”, “PI3K/AKT/mTOR”, “MAPK”, “compensatory signaling”, “adaptive resistance”, “tumor plasticity”, “multitarget inhibitors”, “targeted therapy”, “drug repurposing”, “network medicine”, “anti-HER2 therapy”, “trastuzumab resistance”, “lapatinib resistance”, “endocrine resistance”, “CDK4/6 inhibitors”, “tyrosine kinase inhibitors” and “predictive biomarkers”. We excluded preprints, articles with limited clinical or experimental evidence, unclear methodologies, or non-reproducible results. Articles published between 2008 and 2025 were considered, yielding 122 studies that were critically analyzed and integrated to construct a mechanistic and translational framework of TPBC recurrence.

## 3. Generalities of Triple Positive Breast Cancer (TPBC)

TPBC is a breast cancer subtype characterized by the overexpression of HER2 and the expression of both ER and PR [15]. This subtype of BC has been reported to occur in 10% to 15% of all breast cancer cases, with variability related to population characteristics such as age, as it has been shown to be more common in women aged 50 to 65 years [16].

Non-surgical treatment for TPBC typically consists of chemotherapy combined with HER2-targeted therapy, followed by endocrine therapy when clinically indicated. In the neoadjuvant setting, anti-HER2-based regimens have been associated with pathological complete response rates ranging from approximately 40% to 60%, although outcomes vary according to the therapeutic regimen and patient characteristics [17].

Trastuzumab resistance has been reported in 20% of early-stage BC and up to 70% of metastatic BC cases [18]. Approximately 30% of ER-positive tumors develop de novo resistance to endocrine therapy, while additional mechanisms, such as loss of ER expression, further contribute to treatment failure [19]. Regarding recurrence, TPBC has shown relapse rates of approximately 10% to 30% at 5 years, with early recurrences occurring within 3 to 5 years (related to HER2) and late recurrences occurring after 5 years (associated with the hormonal pathway) [20].

The treatment of BC presents challenges related to adverse effects, particularly those associated with endocrine therapy, which can significantly impact patients’ overall health and quality of life [21].

## 4. Triple-Positive Current Therapies and Limitations

### 4.1. Anti-HER2 Therapies

**Figure 1:**
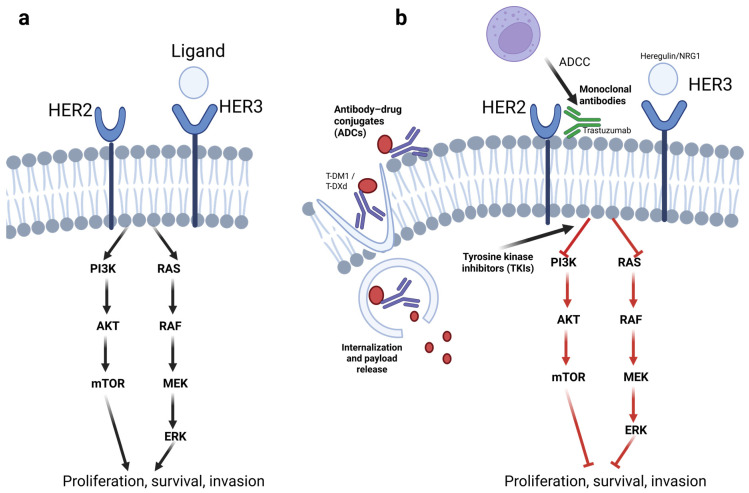
HER2 signaling and major anti-HER2 therapeutic classes in triple-positive breast cancer. (**a**) HER2/HER3 dimerization activates downstream PI3K/AKT/mTOR and RAS/RAF/MEK/ERK signaling pathways, promoting proliferation, survival, and invasion. (**b**) Anti-HER2 therapies include monoclonal antibodies, such as trastuzumab and pertuzumab, which block receptor signaling and may induce antibody-dependent cellular cytotoxicity (ADCC); tyrosine kinase inhibitors (TKIs), such as lapatinib, which inhibit the intracellular kinase domain; and antibody–drug conjugates (ADCs), such as T-DM1 and T-DXd, which bind HER2, undergo internalization, and release a cytotoxic payload. These therapies reduce downstream signaling and tumor-promoting cellular responses. Created in BioRender. Bandala, C. (2026) https://BioRender.com/yx4apj6 (accessed on 22 May 2026).

HER2 is a membrane protein and part of the tyrosine kinase transmembrane receptor family (HER1, HER3, and HER4). It acts as a universal dimerization receptor within the epidermal growth factor receptor (EGFR family [22]. Its overexpression favors the formation of dimers (especially HER2–HER3), autophosphorylation, and sustained activation of pathways such as phosphoinositide 3-kinase (PI3K)/protein kinase B (AKT)/mammalian target of rapamycin (mTOR) and mitogen-activated protein kinase (MAPK)/extracellular signal-regulated kinase (ERK), promoting proliferation, survival, invasion, and therapeutic resistance [23,24]. HER2 plays a critical role in tumor development by promoting proliferation, progression, and cell survival, making it a key target in the development of anti-tumor therapy (Figure 1a) [25].

Anti-HER2 therapies are grouped into three pharmacological categories. The first corresponds to monoclonal antibodies directed against the extracellular domain of the receptor, among which drugs such as trastuzumab and pertuzumab stand out [26,27]. The second group includes small-molecule tyrosine kinase inhibitors, which act on the intracellular domain of the receptor and other members of the HER family [27]. Finally, a third category corresponds to antibody–drug conjugates, which use HER2 as a target for the intracellular release of highly potent cytotoxic agents (Figure 1b) [24].

These strategies are not used in isolation but as part of combined and sequential regimens, with the aim of maximizing HER2 signaling inhibition and delaying the emergence of resistance [27].

#### 4.1.1. Trastuzumab Resistance in TPBC

Trastuzumab is a humanized monoclonal antibody and the first HER2-targeted therapy that blocks dimer formation with EGFR and HER3 (Figure 1b) [22]. This inhibition disrupts HER2 signaling, inducing cell cycle arrest and antibody-dependent cytotoxicity (ADCC) involving the innate immune system, thereby reducing the risk of recurrence, death and metastasis [28,29] by opsonizing HER2-positive tumor cells, facilitating recognition by immune effector cells and increasing the elimination of target cells (Figure 1b). This dual dimension (signal blockade and Fc-mediated immunity) suggests that the therapeutic response may depend on both tumor-intrinsic features and immune-related factors [30].

Despite its benefits, resistance to trastuzumab is inevitable, occurring in up to 50% of cases when combined with chemotherapy and up to 88% with monotherapy [11,27]. Trastuzumab binds to an epitope in the extracellular region domain of the HER2 receptor, producing antitumor effects through complementary pathways [31]. In terms of signaling, trastuzumab inhibits the activation of pathways associated with cell growth and survival (e.g., PI3K/AKT and MAPK pathways) by interfering with HER2 receptor function and reducing proliferative signaling efficiency [32].

Reported mechanisms of resistance to trastuzumab in TPBC include crosstalk between HER2 and hormonal signaling pathways, particularly involving ER/PR mediated upregulation of cyclin-dependent kinase 4 (CDK4) and cyclin D1 [33]; ligand-driven activation of EGFR signaling through factors such as transforming growth factor α (TGFα), heparin-binding epidermal growth factor (EGF), and heregulin [34]; activation of compensatory PI3K/AKT signaling pathways associated with insulin receptor substrate 1 (IRS1) upregulation [35]; and alternative receptor tyrosine kinase signaling [36]. Elevated levels of baculoviral IAP repeat-containing 5 (BIRC5)/survivin, which inhibits apoptosis through multiple signaling pathways, and TNF receptor-associated factor 4 (TRAF4), which stabilizes HER2 by preventing its ubiquitin-mediated degradation, have also been reported as key contributors to trastuzumab resistance [30].

**Figure 2:**
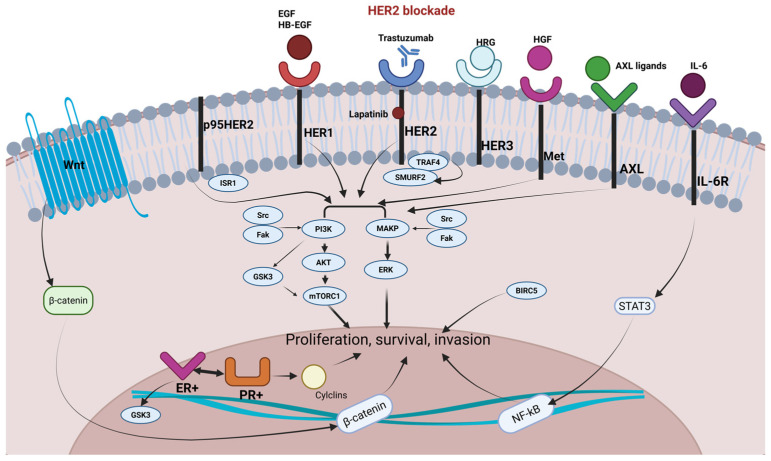
Compensatory signaling pathways associated with trastuzumab and lapatinib resistance in TPBC and related HER2-positive breast cancer models. Trastuzumab and lapatinib inhibit HER2 signaling at the extracellular and intracellular kinase domain levels, respectively. Resistance can arise through receptor-level escape mechanisms, including p95HER2, ligand-driven HER1 and HER3 signaling, Met/HGF activation, AXL-mediated signaling, IL-6/STAT3 activation, β-catenin signaling, and ER/PR pathway crosstalk. These mechanisms converge on PI3K/AKT/mTORC1, MAPK/ERK, Src/FAK, STAT3, NF-κB, β-catenin, and BIRC5, thereby maintaining proliferation, survival, and invasive signaling despite HER2-targeted therapy. The GSK3–mTORC1 route is shown as PI3K-dependent and AKT-independent, in agreement with the mechanism described in the text for lapatinib resistance. Created in BioRender. Bandala, C. (2026) https://BioRender.com/xaeyhqt (accessed on 22 May 2026).

In TPBC, trastuzumab resistance is particularly frequent and clinically relevant (Figure 1). One of the most important mechanisms is related to the activation of downstream signaling pathways (Figure 2), primarily the PI3K/AKT/mTOR pathway [30,36]. Alterations such as functional loss of phosphatase and tensin homolog (PTEN) or activating mutations in phosphatidylinositol-4,5-bisphosphate 3-kinase catalytic subunit alpha (PIK3CA) allow cell survival signals to be maintained even when HER2 is blocked at the receptor level, thus reducing the tumor’s dependence on trastuzumab [37]. Another critical resistance mechanism involves compensatory HER2–HER3 heterodimerization. Although HER3 lacks intrinsic kinase activity, it contains multiple PI3K binding sites that enable sustained activation of the PI3K/AKT pathway, maintaining downstream survival signaling despite HER2 inhibition. This heterodimer is therefore considered a key driver of resistance to HER2-targeted therapies [38]. This mechanism is particularly relevant in TPBC, where the coexistence of multiple signaling pathways promotes functional redundancy and facilitates compensatory survival signaling.

Additionally, the expression of truncated forms of the HER2 receptor (such as p95HER2) has been described. These forms retain intracellular oncogenic activity but lack the extracellular epitope recognized by trastuzumab, preventing effective binding [39]. Finally, in TPBC, crosstalk between HER2 and the estrogen receptor (ER) represents a central resistance mechanism: when HER2 signaling is inhibited, the tumor can shift its dependence toward ER-mediated transcriptional programs, decreasing the effectiveness of anti-HER2 blockade [40].

In early BC, adding trastuzumab to systemic treatment was shown to reduce relapses and improve outcomes compared with chemotherapy alone. A prime example is the HERA program, which evaluated trastuzumab administered after adjuvant chemotherapy, showing benefits in clinical outcomes in HER2-positive patients [41].

Consistently, other adjuvant studies have reinforced that combining chemotherapy with trastuzumab in operable HER2-positive disease improves outcomes compared with regimens without anti-HER2 therapy [22]. In metastatic disease, the role of trastuzumab has been consolidated in both first-line combinations and continuation anti-HER2 strategies [42,43].

#### 4.1.2. Lapatinib Resistance in TPBC

Lapatinib is a reversible tyrosine kinase inhibitor that targets the kinase domain of HER2 and EGFR [44]. It is approved for the treatment of advanced HER2-amplified BC resistant to trastuzumab [45]. However, it shows only a 24% response rate as monotherapy, and patients often develop resistance within six months [25].

Similar to trastuzumab, resistance to lapatinib is linked to increased ER and PR expression [25,37], as well as ER-driven transcriptome enrichment [24]. ER overexpression correlates with AXL receptor tyrosine kinase (AXL) overexpression, which activates the ERK and PI3K/AKT pathways [44], and glycogen synthase kinase 3 (GSK3) overexpression, which in turn activates mTOR complex 1 in a PI3K-dependent and AKT-independent manner (see Figure 2) [24]. HER2 and HER3 have also been implicated in the maintenance of PI3K/AKT signaling despite lapatinib inhibition [46,47]. In addition, alternative signaling pathways, such as Wnt/β-catenin (ingless-related integration site/beta-catenin), which are associated with proliferation and invasion, contribute to lapatinib resistance [48]. Notably, multiple compensatory receptor tyrosine kinases, including nerve growth factor receptor (NGFR), muscle-associated receptor tyrosine kinase (MUSK), vascular endothelial growth factor receptor 1 (VEGFR1), PDGFRα, platelet-derived growth factor receptor β (PDGFRβ), ephrin type-A receptor 2 (EPHA2), and ephrin type-B receptor 2 (EPHB2), are upregulated and collectively contribute to the reactivation of downstream signaling pathways such as extracellular signal-regulated (ERK) [49]. Kinases acting as signaling intermediaries, such as focal adhesion Kinase (Fak) and proto-oncogene, non-receptor tyrosine kinase (Src), are overexpressed in lapatinib-resistant cells and activated by growth factor and adhesion molecule pathways [50].

In TPBC, the interaction with hormonal signaling again plays a central role. Inhibition of HER2 with lapatinib has been shown to induce compensatory activation of the ER, increasing the transcription of ER-dependent genes and promoting cell survival [10,51]. This phenomenon partially explains why dual targeting of HER2 and ER signaling through the combination of lapatinib and endocrine therapy is more effective than monotherapy in TPBC. In this context, resistance to lapatinib is driven not only by compensatory ER signaling but also by additional factors such as limited intracellular drug bioavailability, tumor metabolic adaptation, and clonal heterogeneity, all of which contribute to the progressive loss of therapeutic sensitivity [52,53].

### 4.2. Endocrine Therapies (ER and PR)

In primary breast cancer (PBC), endocrine therapies not only exert direct antiproliferative effects but also influence the efficacy of anti-HER2 therapies through bidirectional crosstalk between ER and HER2 signaling pathways. Inhibition of the ER axis can suppress compensatory signaling mechanisms activated upon HER2 blockade, thereby enhancing the therapeutic response and supporting the rationale for combined treatment strategies [40].

The main classes of endocrine therapies include selective estrogen receptor modulators (SERMs), such as tamoxifen, whose efficacy may be limited by acquired resistance mechanisms [54]; selective estrogen receptor degraders (SERDs), such as fulvestrant, which promote ER degradation and inhibit receptor signaling [55]; and aromatase inhibitors, including letrozole and anastrozole, which suppress estrogen biosynthesis [10,56].

**Figure 3:**
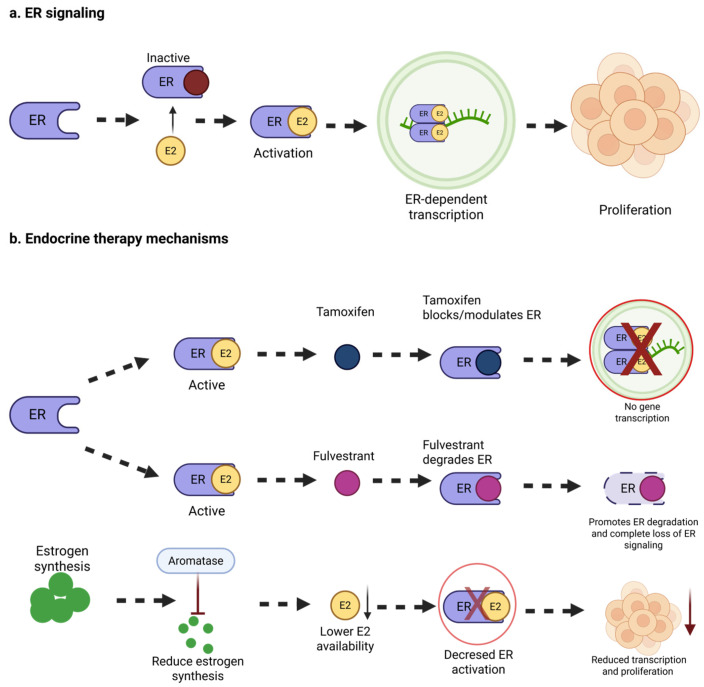
Estrogen receptor signaling and endocrine therapy mechanisms in triple-positive breast cancer. Estradiol (E2) binds to estrogen receptor (ER), promoting receptor activation, nuclear translocation, ER-dependent transcription, and tumor-promoting cellular responses such as proliferation and survival. Endocrine therapies interfere with this axis at different levels: tamoxifen acts as a selective estrogen receptor modulator (SERM) that blocks or modulates ER-mediated transcriptional activity; fulvestrant acts as a selective estrogen receptor degrader (SERD) that promotes ER degradation and suppresses ER signaling; and aromatase inhibitors, such as letrozole and anastrozole, reduce estrogen biosynthesis and decrease E2 availability. Black dashed arrows indicate signaling progression; red T-shaped bars indicate inhibition; red crossed symbols indicate blockade of ER-mediated transcription; downward arrows indicate reduced estrogen levels or cell proliferation. Created in BioRender. Bandala, C. (2026) https://BioRender.com/ms7axp9 (accessed on 1 June 2026).

The hormone receptors ER and PR are transcription factors that regulate genes involved in proliferation, apoptosis, and tumor growth (Figure 3) [25]. Tumors overexpressing ER and PR are primarily treated with ER-targeting therapies such as tamoxifen, fulvestrant, and aromatase inhibitors [57].

#### 4.2.1. Tamoxifen Resistance in TPBC

**Figure 4:**
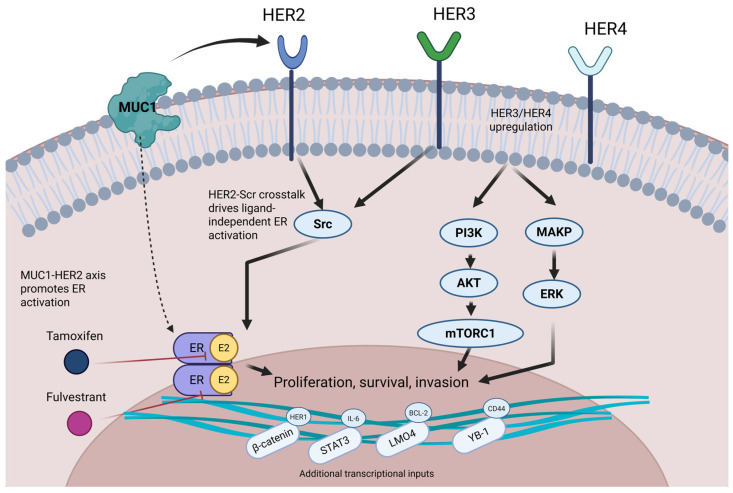
Mechanisms of resistance to endocrine therapy in triple-positive breast cancer (TPBC). Endocrine therapies inhibit estrogen receptor (ER) signaling by modulating the ER via tamoxifen or by degrading the ER via fulvestrant. In TPBC, compensatory signaling can reduce endocrine sensitivity through MUC1-HER2 interaction, HER2-Src crosstalk, and ligand-independent ER activation. Concurrently, HER3/HER4 overexpression can reactivate PI3K/AKT/mTORC1 and MAPK/ERK signaling, maintaining proliferative and survival pathways despite endocrine therapy. These mechanisms converge at the nuclear level by maintaining ER-dependent transcriptional programs, including genes associated with cell cycle progression and survival, ultimately promoting proliferation, survival, and invasion. Created in BioRender. Bandala, C. (2026) https://BioRender.com/e2ft2wy (accessed on 22 May 2026).

Tamoxifen is one of the most effective treatments for ER/PR-positive BC [58]. However, approximately 50% of ER-positive cancers are intrinsically resistant, and 40% of responders eventually acquire resistance [59]. The most described mechanism involves ER–HER2 crosstalk, where HER2 overexpression forms a complex with ER–Src [9,60], acting as a ligand-independent ER activator (Figure 3 and Figure 4). Additionally, when tamoxifen is bound to the ER, prolonged estrogen deprivation triggers a compensatory upregulation of the PI3K/AKT/mTOR survival network [61]. Another mechanism that is activated is related to the activation of tyrosine kinase receptors such as EGFR, insulin-like growth factor 1 receptor (IGF-1R) and fibroblast growth factor receptor (FGFR), which stimulate the PI3K/AKT and MAPK/ERK pathways, decreasing the antagonist effect and dependence on estrogen signaling [10]. Similarly, mucin 1 (MUC1) overexpression stabilizes HER2 and amplifies survival signaling, leading to reduced sensitivity to endocrine therapy [62] and downregulation of ER coactivators such as amplified in breast cancer (AIB1), which restores tamoxifen sensitivity [58]. Overexpression of AIB1 is particularly relevant, as it enhances ER transcriptional activity and can convert tamoxifen from an antagonist to a partial agonist, promoting tumor growth [63]. Low expression of markers such as CXXC4 reduces tamoxifen sensitivity due to inhibition of the Wnt/β-catenin pathway, promoting a more aggressive phenotype since it favors the acquisition of characteristics of tumor stem cells [29].

#### 4.2.2. Fulvestrant Resistance in TPBC

SERDs, such as fulvestrant, induce ER degradation and eliminate its transcriptional function [64]. Fulvestrant has shown efficacy in advanced disease and represents a relevant strategy to counteract ER activation as an escape mechanism in anti-HER2-treated (BC) [25,65]. Resistance mechanisms to fulvestrant include the following: 1. Increased HER3 and HER4 expression (Figure 4), promoting proliferation in TPBC [66,67], and reduced expression of mediator complex subunit 1 (MED1), which restores cell cycle arrest and tumor regression induced by fulvestrant [68], 2. Mutations in ERα (Y537S, Y537N, D538G) confer activity even in the absence of its ligand. Additionally, by promoting the degradation of ERα, it increases its overexpression as a compensatory mechanism [69], 3. Activation of the PI3K/AKT/mTOR pathway, promoting cell proliferation and survival [70], 4. Alternative protumor signaling pathways that reduce estrogen dependence, such as EGFR, IGF-1R, FGFR, and MET [71], 5. Tumor microenvironment signaling mediated by cancer-associated fibroblasts (CAFs), tumor-associated macrophages (TAMs), proinflammatory cytokines (IL-6, TNF-α), and tumor hypoxia can activate alternative survival pathways [72], 6. Hyperactivation of cyclin D1, CDK4, and CDK6 promotes cell proliferation even though ER is blocked [73], 7. Epigenetic changes such as DNA methylation, modified histones, and alterations in microRNAs [74]. Therefore, acute therapeutic strategies suggest combination therapy with fulvestrant with anti-HER2, CDK4/6 inhibitors and PI3K/mTOR inhibitors and other alternative drugs that allow preventing and attenuating resistance mechanisms [75].

## 5. Molecular Mechanisms of Recurrence

Recurrence in TPBC is increasingly recognized as a multifactorial process driven by the interaction of several molecular mechanisms rather than by isolated genetic alterations. Although integrative mechanistic studies specifically focused on TPBC remain limited, available evidence from clinical studies, transcriptomic analyses, and preclinical models of hormone receptor-positive/HER2-positive breast cancer suggests that recurrence is associated with multiple interconnected processes, including the following:

- ER–HER2 signaling redundancy
- PI3K-centered convergence and kinase rewiring
- Therapy-induced stemness and plasticity
- Impaired immune surveillance

Rather than functioning as independent mechanisms, these processes interact dynamically under therapeutic pressure to create a resilient tumor state capable of survival and regrowth. This system-level resilience provides a strong biological rationale for therapeutic strategies aimed at simultaneously targeting multiple interconnected pathways, as discussed in the following section on multi-target drug repurposing.

### 5.1. Adaptive Signaling and PI3K Convergence

The PI3K–AKT–mTOR signaling pathway is a central regulator of cell growth and proliferation in cancer. Moreover, hyperactivation of the PI3K–AKT–mTOR pathway often underlies the development of treatment resistance in cancer [76]. *PIK3CA* (encoding the PI3Kα isoform) is the most frequently mutated gene in ER+/PR+ BC, occurring in up to 40% of cases [77,78]. Receptor tyrosine kinases (RTKs), as well as alterations in *PIK3CA* and its downstream effectors, contribute to the oncogenic activation of the PI3K/Akt/mTOR pathway [79]. Abnormal PI3K activity represents a transforming event during disease progression [80]. The PI3K and ER pathways influence the prognosis of patients with BC [81]. PI3K comprises 8 mammalian isoforms and is a membrane-associated enzyme. These enzymes are categorized into three classes (I–III), with class I PI3Ks being the most relevant to cancer. The p110α subunit, encoded by *PIK3CA*, is the most frequently altered catalytic subunit of PI3K in BC [82]. Several receptor tyrosine kinases, including HER2/ERBB2, EGFR, MET, RET, and VEGFR, transduce extracellular stimuli into intracellular signals and recruit PI3K to the plasma membrane through adaptor proteins such as IRS1 or via RAS (rat sarcoma virus) activation [82]. Upon activation, PI3K-p110α converts phosphatidylinositol-4,5-bisphosphate (PIP2) into phosphatidylinositol-3,4,5-trisphosphate (PIP3), thereby triggering AKT/mTOR signaling [83]. *PIK3CA* mutations have been identified in approximately 40% of advanced HER2-positive BC [84]. Furthermore, HER2 overexpression aberrantly activates the PI3K/AKT signaling pathway in TPBC [85]. Overall, *PIK3CA* mutations are highly prevalent in BC, occurring in approximately 35.7% of tumors and being particularly common in ER-positive disease [86].

### 5.2. TGF-β–Driven Stemness and Residual Disease

Chemotherapy-induced recurrence in BC has been mechanistically linked to autocrine TGF-β signaling–mediated expansion of cancer stem cells (CSCs) [87]. Aurora kinase A (AURKA) acts downstream of TGF-β signaling to reinforce stemness-associated plasticity and chemoresistance, and the combined inhibition of TGF-β and AURKA significantly impairs tumor regrowth [88]. In TPBC, the early loss of TGF-β-mediated growth regulation evolves into a fundamental dysregulation that governs cellular interactions and phenotypes driving invasive disease [10,89]. This pathway promotes epithelial–mesenchymal transition (EMT), inducing cancer stem cell-like properties that enable tumor cells to evade standard therapies, thereby contributing to residual disease and recurrence [90]. EMT plays a significant role in BC initiation, progression, and metastasis [91]. The induction of EMT results from the synergistic action of multiple signaling pathways and molecular regulators, with the TGF-β family acting as a major inducer [92], leading to increased epithelial cell plasticity [90]. TGF-β is widely recognized as a critical factor involved in the acquisition of carcinoma cell plasticity through EMT [90], and its accumulation within the tumor microenvironment (TME) may contribute to therapeutic resistance [93].

### 5.3. Cancer Stem Cells and Phenotypic Plasticity

Phenotypic plasticity is defined as the ability of cells to switch between different phenotypic states without altering their genotype and is widely observed during embryonic development, wound healing, and cancer metastasis [94]. The molecular machinery underlying cancer phenotypic plasticity involves a complex interplay among genetic alterations, lineage-defining transcription factors, epigenetic regulators, metabolic pathways, and crosstalk within the tumor microenvironment [95].

In TPBC, signaling networks involving ER, HER2 overexpression, the PI3K/AKT/mTOR pathway, and MAPK signaling coexist and interact extensively [85]. The interplay among these pathways promotes a highly adaptive tumor phenotype. When one signaling axis is therapeutically inhibited, alternative pathways may become activated, leading to tumor adaptation, intratumoral heterogeneity, and therapeutic resistance [94].

Cancer stem cells (CSCs) are a rare population of tumor cells capable of self-renewal and multilineage differentiation, thereby generating the heterogeneous cellular populations that constitute the tumor mass [96]. CSCs are considered major contributors to intratumoral heterogeneity and exhibit remarkable metabolic plasticity, allowing them to survive under diverse microenvironmental conditions [97].

Closely associated with CSC characteristics, the metastatic process is initiated by cellular dedifferentiation and the acquisition of invasive properties, which enhance the motility and dissemination of cancer cells [98]. CSCs are generally resistant to chemotherapy and radiotherapy; therefore, despite the successful elimination of a substantial proportion of the tumor mass, a residual CSC population may survive and drive cancer recurrence, invasion, metastasis, and therapeutic resistance [99]. CSCs are regulated by a variety of intrinsic and extrinsic factors. Key intrinsic regulators include genetic, epigenetic, and metabolic programs, whereas extrinsic regulators involve interactions with the tumor microenvironment, including niche-derived factors and immune system components [97,99].

### 5.4. Immune Evasion and Microenvironmental Remodeling

To evade immune surveillance, TPBC remodels its tumor microenvironment (TME), creating an immunosuppressive milieu through the recruitment and activation of immunoregulatory cells, such as M2-polarized macrophages and regulatory T cells (Tregs), as well as by disrupting immune checkpoint signaling pathways [100]. TPBC can evade immune destruction through immune checkpoint pathways, particularly the PD-1 (programmed cell death protein 1)/PD-L1 (programmed death-ligand 1) axis, and through the secretion of immunosuppressive molecules. These mechanisms suppress T-cell activity, allowing tumor cells to proliferate while escaping immune recognition [101]. In TPBC, Shekhar et al. demonstrated using a three-dimensional cell–cell interaction model that normal fibroblasts (NFs) inhibited estrogen-induced tumor cell growth, whereas CAFs produced abundant estrogens that promoted the malignant transformation of the normal mammary epithelial cell line MCF10A and the premalignant mammary epithelial cell line EIII8 [102]. TME plays a critical role in TPBC progression, metastasis, and therapeutic resistance [1]. These interactions facilitate tumor growth, immune evasion, and the establishment of a microenvironment that supports cancer cell survival and proliferation [103]. The TME comprises both cellular and non-cellular components surrounding malignant tumor cells [104]. Cellular constituents include mesenchymal and hematopoietic cells. Mesenchymal cells comprise fibroblasts, myofibroblasts, adipocytes, endothelial cells, and mesenchymal stem cells (MSCs), whereas hematopoietic cells include lymphoid populations such as T cells, B cells, dendritic cells, mast cells, and natural killer (NK) cells, as well as bone marrow-derived cells including TAMs, neutrophils, and myeloid-derived suppressor cells (MDSCs) [105].

The TME also encompasses critical metabolic conditions, including pH, oxygen tension (PO_2_), glucose, glutamine, and lactate concentrations, as well as chemical mediators such as nitric oxide (NO) [100]. Interactions among these TME components collectively promote tumor initiation, progression, invasion, and metastasis while influencing therapeutic responses [106]. Drug resistance in TPBC arises from multifactorial mechanisms involving coordinated signaling networks and complex interactions with multiple components of the TME. However, despite increasing interest in this field, studies specifically addressing the TPBC microenvironment remain limited [100].

## 6. Repurposing Multi-Target Inhibitors

Drug repurposing involves identifying new therapeutic applications for existing drugs. This approach reduces costs, shortens development timelines, and leverages established safety profiles. In TPBC, multi-target inhibitors may simultaneously modulate hormone receptor and growth factor receptor signaling, overcoming compensatory mechanisms. Repurposed drugs can simultaneously target multiple nodes within the oncogenic signaling network, including receptor-level, intracellular, and metabolic pathways, thereby reducing tumor adaptability and recurrence potential.

Examples include CDK4/6 inhibitors combined with HER2 blockade, PI3K/AKT/mTOR dual inhibitors, and AXL inhibitors targeting lapatinib resistance. Non-oncological drugs such as metformin and statins have also been investigated for repurposing due to their effects on tumor metabolism and survival pathways. In silico and in vitro studies, as well as ongoing clinical trials, support their potential in TPBC treatment.

Table 1 summarizes the key resistance mechanisms discussed in the study, the corresponding therapeutic strategies, the proposed target molecules, and the limitations of the evidence. These strategies should be understood as approaches to modulating recurrence-associated mechanisms and not as established therapies for recurrence prevention in TPBC (Table 1).

### 6.1. Systems Biology, Network Medicine, and AI-Driven Drug Repurposing

Unlike conventional target-centered approaches, systems biology and network medicine evaluate diseases as interconnected molecular networks rather than isolated signaling pathways. AI-assisted algorithms can integrate genomic, transcriptomic, proteomic, pharmacological, and clinical data sets to identify highly connected nodes, network bottlenecks, and pathway crosstalk mechanisms that may not be evident through traditional analyses. These approaches enable the prioritization of proteins, pathways, and drug combinations based on their network influence rather than solely on differential expression or individual biological functions. Consequently, candidate targets are selected according to their predicted capacity to modulate multiple disease-associated processes simultaneously.

Recent studies in network medicine and GenAI-assisted analyses have significantly accelerated the identification of candidate multi-target agents for complex cancers. For example, a systems biology framework integrating clinical trial data, signaling pathway databases (e.g., the KEGG breast cancer module), and large-scale literature mining identified numerous repurposable drugs with potential cross-pathway activity [107]. In that analysis, among 46 BC-associated pathways, 38 were covered by at least two drugs, including HER2-related signaling (hsa:2064), which exhibited more than 100 drug–pathway interactions. These findings suggest that agents such as metformin, statins, CDK4/6 inhibitors, and PI3K/AKT/mTOR dual inhibitors may simultaneously modulate hormone receptor and growth factor receptor signaling pathways. However, these network-based observations should be considered hypothesis-generating and do not constitute direct evidence of therapeutic efficacy or recurrence prevention in TPBC, which requires further experimental and clinical validation [107].

Building on these systems-level observations, recent studies have proposed multitarget drug design as a promising paradigm in oncology, emphasizing the perturbation of interconnected signaling networks involved in therapeutic resistance and disease recurrence [108]. Within this framework, TPBC represents a biologically complex context characterized by the coexistence of hormonal and HER2-driven signaling programs. Consequently, multi-target approaches capable of modulating multiple redundant pathways may warrant further investigation in this subtype. In breast cancer, these interconnected networks frequently involve HER2/EGFR, PI3K/AKT/mTOR, and Janus Kinase/signal transducer and activator of transcription (JAK/STAT) axes, providing a rationale for the evaluation of rationally designed multi-target inhibitors or the repurposing of approved multikinase TKIs, such as lapatinib, sorafenib, and regorafenib. Nevertheless, the therapeutic value of these approaches in TPBC remains to be established through preclinical and clinical studies.

### 6.2. Targeting Compensatory Signaling and Receptor Cross-Talk

Resistance to anti-HER2 therapies arises from complex molecular adaptations, including activation of PI3K/AKT/mTOR, AXL, and CDK4/6 signaling, as well as epithelial–mesenchymal transition (EMT) and immune evasion mechanisms. As reviewed by [109], novel therapeutic strategies combining targeted inhibitors (e.g., alpelisib, everolimus, palbociclib) with anti-HER2 agents have shown significant promise in overcoming these adaptive networks. Such multitarget approaches are particularly relevant for TPBC, where concurrent ER and HER2 signaling promote metabolic and proliferative redundancy. In this context, repurposed agents such as metformin, statins, and propranolol may further complement these regimens by modulating convergent pathways, including mTOR, NF-κB, and AXL-dependent signaling, offering a cost-effective strategy to prevent recurrence and therapeutic resistance.

Consistently, the receptor tyrosine kinase AXL has emerged as a key driver of resistance to HER2 blockade in BC, including TPBC. Ligand-independent AXL–HER2 heterodimerization sustains PI3K/AKT and MAPK signaling, promoting EMT, invasion, and recurrence [110]. Importantly, inhibition of AXL restores sensitivity to trastuzumab and lapatinib in preclinical and patient-derived xenograft (PDX) models, achieving marked tumor regression. Given that several AXL inhibitors—such as bemcentinib, foretinib, cabozantinib, and TP-0903—are clinically available or in advanced trials, their repurposing as multi-target agents represents a promising strategy to overcome HER2 resistance and prevent recurrence in TPBC [111].

### 6.3. Metabolic Rewiring and Epigenetic Plasticity as Multitarget Vulnerabilities

Metabolic adaptation represents a major driver of resistance in HER2-positive and TPBC tumors. Metformin has emerged as a prototypical multi-target repurposed drug capable of bridging metabolism, signaling, and immune regulation in BC. Through activation of activated protein kinase (AMPK) and inhibition of the PI3K/AKT/mTOR axis, metformin suppresses proliferation, EMT, and cancer stem cell renewal while enhancing the efficacy of anti-HER2 and endocrine therapies. Its pleiotropic actions extend to microRNA modulation, epigenetic remodeling, and immune activation, making it particularly relevant for TPBC, where metabolic and signaling plasticity drive recurrence [112].

Emerging clinical evidence provides a rationale for further investigation of metformin in HER2-positive BC. A comprehensive scoping review of 40 randomized controlled trials reported heterogeneous outcomes; however, subgroup analyses from a major clinical trial suggested improved overall and disease-free survival among HER2-positive patients receiving metformin, a benefit that was not consistently observed across other BC subtypes. Mechanistically, metformin has been shown in preclinical studies to modulate the HER2/EGFR, PI3K/AKT/mTOR, NF-κB, STAT3, and TGF-β signaling pathways. These observations support its potential as a multitarget therapeutic candidate capable of influencing several biological processes associated with proliferation, therapeutic resistance, and tumor progression. Nevertheless, the clinical relevance of these effects in TPBC remains uncertain, as current evidence is largely derived from preclinical studies, retrospective analyses, or subgroup observations rather than prospective clinical trials specifically designed for TPBC patients [113].

At the transcriptional level, Zhang [114] identified the epigenetic plasticity of CSCs as a major therapeutic bottleneck underlying drug resistance and recurrence. Reversible reprogramming through DNA methylation, histone modification, and non-coding RNA regulation enables tumor cells to switch between differentiated and quiescent states, maintaining heterogeneity and survival after targeted or endocrine therapy. In BC, this adaptive epigenetic landscape intersects HER2 and ER signaling, reinforcing stemness, immune evasion, and metabolic resilience. Accordingly, histone deacetylase (HDAC) and DNA methyltransferase (DNMT) inhibitors represent key multitarget strategies capable of restoring differentiation and resensitizing tumors to receptor-directed therapies.

### 6.4. Repurposed Non-Oncologic Agents as Multitarget Adjuvants

Several non-oncologic drugs have demonstrated strong potential as multitarget agents in TPBC. Statins, as comprehensively discussed by Tripathi [115], exemplify repurposed multi-target drugs by modulating cholesterol biosynthesis, protein prenylation, and oncogenic signaling networks, including PI3K/AKT/mTOR, extracellular signal-regulated kinase (ERK), and STAT3. Their pleiotropic effects extend to indirect epigenetic and immune regulation, positioning statins as low-cost adjuvants capable of enhancing standard treatments such as trastuzumab or doxorubicin in HER2-positive and TPBC. Mechanistically, inhibition of 3-hydroxy-3-methylglutaryl-coenzyme A (HMG-CoA) reductase diminishes prenylation of RAS (rat sarcoma), RHO (Ras Homolog), and RAC (ras-related C3 botulinum toxin substrate), thereby indirectly modulating HER2- and ER-dependent signaling [116,117].

Similarly, the nonselective β-blocker propranolol has emerged as a promising multitarget repurposed agent. By antagonizing β1/β2-adrenergic signaling, propranolol inhibits pathways involved in proliferation, angiogenesis, and resistance, including PI3K/AKT, MAPK (mitogen-activated protein kinase), and HIF-1α (hypoxia-inducible factor 1-alpha)/VEGF. In TPBC, where hormonal and growth factor signaling intersect with adrenergic stress responses, propranolol may synergize with anti-HER2 and endocrine therapies to prevent recurrence and enhance treatment efficacy [118].

The Hedgehog (Hh) signaling pathway also plays a pivotal role in therapeutic resistance and tumor microenvironment remodeling in breast cancer. Aberrant Hh activation sustains EMT, stemness, and immune evasion by reprogramming tumor-associated fibroblasts, macrophages, and regulatory T cells. Crosstalk with PI3K/AKT/mTOR, TGF-β (transforming growth factor-beta), NF-κB (nuclear factor kappa-light-chain-enhancer of activated B cells), and Wnt/β-catenin further amplifies proliferative and anti-apoptotic signaling, contributing to recurrence in TPBC. Accordingly, multitarget inhibitors such as itraconazole, vismodegib, or natural GLI (glioma-associated oncogene) inhibitors such as genistein, curcumin, and sinomenine have shown promise in disrupting this complex signaling network [119,120].

### 6.5. Combinatorial Repurposing and Transcriptional Network Control

Finally, Liu [121] provided a compelling example of rational combinatorial repurposing through the C3 regimen (metformin, simvastatin, and digoxin), which suppresses tumor growth by jointly inhibiting the transcriptional hubs PDX1 and BIRC5 (survivin). By integrating metabolic (AMPK/mTOR), lipidomic (mevalonate–RAS/RHO), and ionic (Na^+^/K^+^-ATPase) perturbations, this combination reactivates apoptotic networks and overcomes resistance. Although developed in pancreatic ductal adenocarcinoma, this multitarget transcriptional strategy is highly relevant for TPBC, where overlapping survival pathways—HER2, PI3K/AKT/mTOR, and survivin—drive therapeutic resistance and recurrence.

Collectively, systems-based drug repurposing approaches underscore a cost-effective and biologically grounded strategy for identifying multi-target therapeutic candidates in TPBC. By integrating network medicine, AI-guided discovery, and rational combination therapies, these approaches may facilitate the development of future treatment strategies targeting multiple mechanisms associated with therapeutic resistance and recurrence. Nevertheless, further experimental and clinical validation is required to establish their therapeutic efficacy and clinical relevance [122].

The clinical translation of multi-target repurposed drugs faces challenges, including regulatory approval, intellectual property issues, and optimization of combination regimens. However, ongoing clinical trials investigating combinations of HER2 inhibitors with CDK4/6 or PI3K pathway inhibitors highlight the feasibility of these approaches. Personalized medicine frameworks will be essential to match patients with effective multi-target therapies based on biomarker profiling.

### 6.6. Current Limitations and Future Perspectives

Despite the promising rationale for multi-target drug repurposing in TPBC, several limitations remain. First, clinical evidence supporting many repurposed agents is heterogeneous, with benefits often derived from subgroup analyses, retrospective studies or preclinical investigations rather than prospective TPBC-specific clinical trials. Second, the pharmacodynamic complexity of TPBC, including HER2–ER crosstalk, pathway redundancy, and adaptive resistance mechanisms, may limit the long-term efficacy of single-agent interventions. Finally, most available studies have been conducted in HER2-positive or hormone receptor-positive/HER2-positive populations without specifically stratifying patients according to TPBC status. Consequently, the clinical relevance of many proposed therapeutic strategies remains uncertain and requires validation in dedicated TPBC-focused preclinical and clinical studies.

## 7. Conclusions

TPBC is a breast cancer subtype that, despite the availability of targeted therapies, remains a significant clinical challenge. This is due to the complexity of its molecular landscape, which promotes recurrence and therapeutic resistance, as well as barriers related to treatment accessibility, cost, and adherence. Although drug repurposing has been explored primarily in triple-negative and other aggressive breast cancer subtypes, the evidence reviewed here suggests that TPBC represents a relevant setting in which drug repurposing approaches, particularly those involving multi-target inhibitors, warrant further investigation. By simultaneously targeting multiple pathways implicated in resistance and recurrence, these strategies may provide a valuable framework for future therapeutic development; however, their clinical utility remains to be established through experimental and clinical validation.

## Figures and Tables

**Table 1:** Potential therapeutic strategies targeting mechanisms associated with recurrence in TPBC.

Recurrence-Associated Mechanism	Candidate Strategy	Main Target	Evidence Basis and Key Limitation
ER/HER2 crosstalk	Endocrine therapy + anti-HER2 therapy: tamoxifen, fulvestrant, aromatase inhibitors, trastuzumab, pertuzumab, lapatinib	Blocks ER signaling and HER2-driven compensatory activation	Supported by evidence regarding HR+/HER2+ and HER2+ breast cancer [9,10,26,41,52,61,62,64]. Validation regarding recurrence in TPBC remains limited.
PI3K/AKT/mTOR and MAPK/ERK reactivation	PI3K/mTOR inhibitors and combination strategies: alpelisib, everolimus, anti-HER2 combinations	Targets downstream survival and proliferative signaling after HER2 blockade	Supported by literature on HER2+ and TPBC-related resistance [31,37,38,76]. The benefit depends on biomarkers, toxicity, and patient selection.
Cell-cycle escape	CDK4/6 inhibitor-based combinations, especially with endocrine and/or anti-HER2 therapy.	Targets Cyclin D–CDK4/6–RB-driven proliferation	Evidence comes mainly from HR+ or HR+/HER2+ contexts [34,63,76]. TPBC-specific recurrence data remain limited.
AXL-mediated escape and EMT	AXL inhibitors combined with trastuzumab or lapatinib; examples: bemcentinib, foretinib, cabozantinib, TP-0903	Blocks AXL-driven PI3K/AKT, MAPK, EMT, invasion and HER2 therapy escape	Predominantly preclinical and PDX evidence [45,77,78]. No established AXL-targeted strategy for TPBC.
Metabolic and stress-adaptive signaling	Repurposed adjunctive agents: metformin, statins, propranolol	Modulates AMPK/mTOR, mevalonate-RAS/RHO/RAC signaling, β-adrenergic signaling, PI3K/AKT, MAPK, HIF-1α/VEGF	Mixed clinical, observational, and preclinical evidence [79,80,81,82,83,84,85]. Not specifically designed to prevent TPBC recurrence.
Stemness, EMT and anti-apoptotic survival programs	Exploratory agents: itraconazole, vismodegib, GLI inhibitors, digoxin as part of the C3 regimen concept	Targets Hedgehog/SMO/GLI, Wnt/β-catenin, BIRC5/survivin and apoptosis-resistance programs	Mainly preclinical or extrapolated evidence [86,87,88]. The evidence on digoxin/C3 was extrapolated from pancreatic cancer research and should be interpreted with caution.

## Data Availability

No new data were created or analyzed in this study. Data sharing is not applicable to this article.

## Abbreviations

The following abbreviations are used in this manuscript:

TPBC	Triple-Positive Breast Cancer
ER	Estrogen Receptor
PR	Progesterone Receptor
HER2 = EGFR2	Human Epidermal Growth Factor Receptor 2
BC	Breast cancer
NCCN	National Comprehensive Cancer Network
NCBI	National Center for Biotechnology Information
EGFR = HER1	Epidermal Growth Factor Receptor
HER3	Human Epidermal Growth Factor Receptor 3
HER4	Human Epidermal Growth Factor Receptor 4
PI3K	phosphatidylinositol 3-kinase
AKT	a serine/threonine protein kinase
mTOR	[Ser/Thr kinase] mechanistic target of rapamycin
MAPK	mitogen-activated protein kinases
ERK	Extracellular Signal-Regulated Kinase
ErbB	Erythroblastic Leukemia Viral Oncogene Homolog [family]
ADCC	Antibody-dependent cytotoxicity
Fc-mediated immunity	Constant fragment [of antibodies that mediates downstream effector functions via its interaction with Fc-receptors on (innate) immune cells]
CDK4	Cyclin-Dependent Kinase 4
TGF-α	Transforming Growth Factor Alpha
EGF	Epidermal Growth Factor
Met	methionine
BIRC5	baculoviral inhibitor of apoptosis repeat-containing 5
TRAF4	Tumor Necrosis Factor Receptor-Associated Factor 4
PTEN	Phosphatase and Tensin homolog
PIK3CA	gene encodes for the p110 alpha (p110α) protein, catalytic subunit of PI3K
CD55	Decay-accelerating factor (CD55 = DAF)
CD59	[Protective protein]
Wnt3	Wnt refers to a family of secreted signaling proteins involved in various biological processes, particularly in cell communication and development. The name “Wnt” is a portmanteau derived from “Wingless”, a gene from the fruit fly Drosophila, and “Int-1”, a proto-oncogene from mice.
PDGFRA	gene encodes for PDGFRα
PDGFRα	platelet-derived growth factor receptor alpha
MUC1-C	C-terminal subunit for MUC1 protein
IGF-1R	Insulin-like Growth Factor 1 Receptor
P85β	regulatory subunit of PI3K
P110	isoforms of the class-1A PI3K signaling pathway
Yes1	non-receptor protein tyrosine kinase
Src	non-receptor protein tyrosine kinase
OXPHOS	Oxidative Phosphorylation
NDUFA4L2	NADH Dehydrogenase (Ubiquinone) 1 Alpha Subcomplex 4-Like 2
ROS	Reactive Oxygen Species
LMO4	LIM Domain Only 4
YB-1	Y-box Binding Protein 1
CD44	Cluster of Differentiation 44
IL6	Interleukin-6
BCL-2	B-cell Lymphoma 2
P95her2	Truncated HER2 receptor (p95HER2)
HERA program	HERceptin Adjuvant trial
AXL	AXL Receptor Tyrosine Kinase
GSK3	Glycogen Synthase Kinase 3
mTOR-C1	mTOR complex 1
Wnt	Wingless/Integrated
NFGR	Nerve Growth Factor Receptor
MUSK	Muscle-Specific Kinase
VEGFR1	Vascular Endothelial Growth Factor Receptor 1
PDGFRA	Platelet-Derived Growth Factor Receptor Alpha
PDGFβ	Platelet-Derived Growth Factor Beta
EPHA2	Ephrin Type-A Receptor 2
EPHB2	Ephrin Type-B Receptor 2
Fak = FAK1	Focal adhesion kinase 1
PBC	primary breast cancer
SERMs	Selective Estrogen Receptor Modulators
MUC1	Mucin-1
AIB1	Amplified in Breast Cancer 1 (SRC-3)
SERD	Selective Estrogen Receptor Degrader
CXXC4	CXXC Finger Protein 4
PBCC	primary basal cell carcinoma
MED1	Selective Estrogen Receptor Degrader
Eph	Eph receptors
AKT1	AKT Serine/Threonine Kinase 1
CSC	cancer stem cells
TGF-β	Transforming Growth Factor Beta
STAT3	Signal transducer and activator of transcription 3
NF-kB	Nuclear Factor kappa B
SMAD	Transcription factors proteins for TGF-β. SMAD is a portmanteau from SMA (small body size) and MAD (Mothers Against Decapentaplegic).
AURKA	Aurora kinase A
TrkB	Tropomyosin Receptor Kinase B
SOX2	SRY-Box Transcription Factor 2
CD24	Cluster of Differentiation 24
MHC	Major Histocompatibility Complex
HLA	Human Leukocyte Antigen
CD4/6	Cyclin-Dependent Kinases 4/6
AI	artificial intelligence
KEEG	Kyoto Encyclopedia of Genes and Genomes (KEGG)
hsa:2064	Human gene identifier (ERBB2/HER2)
JAK	Janus Kinase
TKI	tyrosine kinase inhibitors
EMT	epithelial–mesenchymal transition
PDX	Patient-Derived Xenograft
TP-0903	TP-0903 (drug candidate)
RNA	Ribonucleic acid
HDAC	histone deacetylase
DNA	Deoxyribonucleic Acid
DNMT	DNA methyltransferase
HMG-CoA	3-Hydroxy-3-Methylglutaryl-Coenzyme A
RAS	Rat Sarcoma Virus Oncogene
RHO	Ras Homologous Protein
RAC	Ras-related C3 botulinum toxin substrate
GLI	Glioma-associated Oncogene
Hh	Hedgehog
ATPase	Adenosine Triphosphatase
AMPK	Adenosine Monophosphate Kinase
